# Editorial: Mentalization in the psychosis continuum: current knowledge and new directions for research and clinical practice

**DOI:** 10.3389/fpsyt.2024.1447937

**Published:** 2024-06-26

**Authors:** George Salaminios, Neus Barrantes-Vidal, Patrick Luyten, Martin Debbané

**Affiliations:** ^1^ Research Department of Clinical, Educational and Health Psychology, University College London, London, United Kingdom; ^2^ Research Department, British Association for Counselling and Psychotherapy, Lutterworth, United Kingdom; ^3^ Department of Clinical and Health Psychology, Autonomous University of Barcelona, Barcelona, Spain; ^4^ CIBER de Salud Mental, Instituto de Salud Carlos III, Madrid, Spain; ^5^ Faculty of Psychology and Educational Sciences, KU Leuven, Leuven, Belgium; ^6^ Developmental Clinical Psychology Research Unit, Faculty of Psychology and Educational Sciences, University of Geneva, Geneva, Switzerland

**Keywords:** mentalizing, psychosis, clinical high risk (CHR) for psychosis, mentalization based treatment, schizotypy, schizophrenia, early intervention psychosis, psychotherapy

Imbalances in mentalizing – the capacity to envisage mental states in oneself and others - have consistently been associated with symptomatic and functional outcomes in people with psychosis (1), as well as with the transition to clinical psychosis among those who are at increased risk (2). Recently, applications of mentalization-based therapy (MBT) for individuals in the psychosis spectrum have been developed and empirically evaluated (3, 4). Given the increasing interest in mentalizing as a treatment target in psychosis, the aim of this research topic is to provide a synthesis of current knowledge and new perspectives concerning the potential role of mentalizing across the psychosis spectrum.

The first group of papers in this research topic present new conceptual approaches and empirical studies exploring the role of mentalizing dysfunction and the application of MBT in individuals diagnosed with clinical psychosis. Weijers et al. compared the effectiveness of MBT in improving mentalizing abilities between patients with schizophrenia and patients with a diagnosis of borderline personality disorder (BPD). Their findings show that patients with BPD reported significantly more improvement across a range of mentalizing facets following MBT compared to patients with schizophrenia who also received MBT. Interestingly, patients with schizophrenia who received MBT showed significantly more improvement only on one mentalizing dimension compared to patients with schizophrenia who received treatment as usual. In accordance with findings from a previous RCT (4), these findings illustrate the relevance of MBT to the treatment of psychosis, but also stress the importance of tailoring MBT interventions to better meet the needs of this patient group.

Indeed, a series of conceptual papers in this research topic propose technical adaptations to MBT, with a particular emphasis on the patient-therapist relationship, to address some of the unique challenges that people with psychosis face. Bröcker et al., draw on the psychoanalytic work of Stavros Mentzos (5) to highlight the importance of utilizing “implicit” techniques during the early phases of MBT to sustain a tolerable therapeutic relationship that may better support patients with psychosis to contain unrepresented anxieties pertaining to interpersonal closeness and distance. The authors argue that when working with psychotic patients, an implicit focus on regulating interpersonal contact within the therapeutic relationship should always underpin more explicit or “reflective” MBT techniques. In a similar vein, but this time drawing on Friston’s “free energy” theory (6) and Gergely’s theory on self-agency development (7) Sanz et al. propose a modified MBT approach to support people suffering with enduring forms of psychosis. Their approach also shifts the therapeutic focus away from reflective processes and towards sustaining a “predictable” (in Friston’s terms) dyadic relationship aiming to foster epistemic trust and strengthen the patient’s sense of agency. In their perspective article, Parkinson et al. discuss how combining a group MBT and art therapy approach may support individuals diagnosed with first-episode psychosis to reflect on experiences and emotions that may otherwise be subject to avoidance. Their paper documents this approach based on their experiences with a combined art therapy and mentalization-based psychoeducation group course for people with first-episode psychosis delivered within an Early Intervention for Psychosis service in the UK.

The next two papers focus on metacognition, a construct that conceptually overlaps with mentalizing and captures the ability to synthesize mental knowledge into complex narratives of self and others (8). Salvatore et al. present a case report illustrating the role of clinical supervision in supporting a therapist’s understanding of aspects of her own personal history and how these were enacted in her work with a young woman with psychosis. The authors discuss how supervision strengthened the therapist’s metacognitive capacities, enabling her to tune in to her patient’s painful emotional experiences, and how these may have fostered therapeutic change. A study by Montemagni et al. explores the complex relationships between conceptual disorganization and metacognition in a sample of outpatients with schizophrenia. Their findings show that conceptual disorganization differentially impacts different metacognitive domains and mediates the effects of neurocognitive difficulties on metacognition.

The second group of papers in this research topic focus on the role of mentalizing and the application of MBT in the early stages of the psychosis continuum, that is, among people who are at increased risk for psychosis. Nonweiler et al. explore the complex associations between childhood adversity, mentalizing and psychotic features (i.e. schizotypal traits and psychotic-like experiences) in a large non-clinical adult sample. The authors show that dysfunctions in understanding one’s own mental states (i.e., mentalizing with regard to the self) mediated the association between childhood adversity and non-clinical psychotic features. Moving further along the psychosis continuum, a study by Salaminios et al. explored the associations between schizotypal personality traits, self-reported mentalizing and clinical high risk for psychosis (CHR-P) as assessed in terms of perceptive and cognitive symptoms. Their study showed that schizotypal traits and mentalizing impairments during adolescence and young adulthood were associated, both independently and through their interactions, with early symptomatic signs of CHR-P. The findings from these two latter studies provide further evidence for the assumption that mentalizing may play an important role in determining early trajectories of psychosis expression, thus highlighting its relevance for early prevention and intervention.

Consistent with these assumptions, Dangerfield and Brotnow Decker focus on early intervention in the domain of psychosis and present outcome results from an innovative MBT-based home-treatment program for high-risk youths on the psychosis spectrum that have experienced difficulties in engaging with other forms of psychotherapeutic treatment. Their findings suggest that mentalization-based interventions may foster engagement with treatment resulting in clinically meaningful changes and functional recovery in young people at high risk for psychosis. In the last paper focusing on the pre-clinical stages of psychosis, De Salve et al. present a systematic review of previous research exploring how Theory of Mind (ToM), reflective functioning and metacognitive beliefs relate to state and trait risk for psychosis. Their review suggests that low reflective functioning and the presence of maladaptive metacognitive beliefs are associated with CHR-P symptoms and schizotypal traits in non-clinical individuals, while evidence concerning the association between ToM and psychotic symptoms in non-clinical samples appears to be more mixed.

The final group of papers in this research topic reflect on the future of interventions with a mentalizing focus for individuals with psychosis and related conditions. Gussmann et al. present an empirically-based systematic approach (i.e. intervention mapping) for the development of a clinical intervention that specifically addresses the needs of inpatients during the acute phase of psychosis by targeting metacognitive deficits. Finally, Costa-Cordella et al., discuss how MBT approaches to psychosis (1) may also be applied to the understanding of autism-spectrum disorders.

In sum, the innovative papers that are part of this research topic capture a broad range of contemporary approaches that open up promising directions for basic research in the area and have the potential to inform clinical practice to support meaningful therapeutic outcomes for people suffering with psychosis and those at increased risk. As a next step, we want to encourage research that will empirically evaluate the efficacy and effectiveness of adapted MBT interventions for psychosis, as well as process-outcome research that will test assumptions about potential mechanisms of change in MBT. Finally, to support early intervention, there is a need for longitudinal studies that will explore the associations of mentalizing with other psychosocial and neurobiological risk factors for psychosis during the critical developmental period spanning from adolescence to young adulthood.

## Author contributions

GS: Conceptualization, Writing – original draft, Writing – review & editing. NB-V: Conceptualization, Writing – review & editing. PL: Conceptualization, Writing – review & editing. MD: Conceptualization, Writing – review & editing.
